# A 2 Week Cross-over Intervention with a Low Carbohydrate, High Fat Diet Compared to a High Carbohydrate Diet Attenuates Exercise-Induced Cortisol Response, but Not the Reduction of Exercise Capacity, in Recreational Athletes

**DOI:** 10.3390/nu13010157

**Published:** 2021-01-06

**Authors:** Rieneke Terink, Renger F. Witkamp, Maria T. E. Hopman, Els Siebelink, Huub F. J. Savelkoul, Marco Mensink

**Affiliations:** 1 Division of Human Nutrition and Health, Wageningen University & Research (WUR), 6700 AH Wageningen, The Netherlands; renger.witkamp@wur.nl (R.F.W.); els.siebelink@wur.nl (E.S.); marco.mensink@wur.nl (M.M.); 2 Department of Physiology, Radboud University Nijmegen, 6525 GA Nijmegen, The Netherlands; Maria.Hopman@radboudumc.nl; 3 Cell Biology and Immunology Group, Wageningen University & Research (WUR), 6700 AH Wageningen, The Netherlands; huub.savelkoul@wur.nl

**Keywords:** cortisol, ketones, s-IgA, exercise, low carbohydrate diet

## Abstract

Low carbohydrate, high fat (LCHF) diets are followed by athletes, but questions remain regarding effects of LCHF on metabolic adaptation, exercise-induced stress, immune function and their time-course. In this cross-over study, 14 recreational male athletes (32.9 ± 8.2 years, VO2max 57.3 ± 5.8 mL/kg/min) followed a two week LCHF diet (<10 En% carbohydrates (CHO), ~75En% Fat) and a two week HC diet (>50 En% CHO), in random order, with a wash-out period of >2 weeks in between. After 2 days and 2 weeks on either diet, participants performed cycle ergometry for 90 min at 60%W_max_. Blood samples for analysis of cortisol, free fatty acids (FFA), glucose and ketones, and saliva samples for immunoglobin A (s-IgA) were collected at different time points before and after exercise. The LCHF diet resulted in higher FFA, higher ketones and lower glucose levels compared to the HC diet (*p* < 0.05). Exercise-induced cortisol response was higher after 2 days on the LCHF diet (822 ± 215 nmol/L) compared to 2 weeks on the LCHF diet (669 ± 243 nmol/L, *p* = 0.004) and compared to both test days following the HC diet (609 ± 208 and 555 ± 173 nmol/L, both *p* < 0.001). Workload was lower, and perceived exertion higher, on the LCHF diet compared to the HC diet on both occasions. A drop in s-IgA following exercise was not seen after 2 days on the LCHF diet, in contrast to the HC diet. In conclusion, the LCHF diet resulted in reduced workload with metabolic effects and a pronounced exercise-induced cortisol response after 2 days. Although indications of adaptation were seen after 2 weeks on the LCHF diet, work output was still lower.

## 1. Introduction

Chronic or periodized low carbohydrate, high fat (LCHF) dietary strategies have been applied in sports for several decades. More recently, the interest among some athletes appears to be on the rise again due to the alleged positive effects of ‘ketogenic’ LCHF (K-LCHF) diets and(or) ketone bodies in general [1]. Typically, K-LCHF diets deliver less than 5% of their energy from carbohydrate (CHO) and more than 75% from fat, corresponding to roughly <50 g/day CHO for most athletes [1]. The more general term (non-ketogenic) LCHF is typically used for diets with <15–20 En% from CHO.

It has been shown that (K-)LCHF diets can increase the transport, uptake and beta-oxidation of fat in muscle. In addition, studies have demonstrated an enhanced activation of some enzymes and mediators involved in adaptation to endurance training during situations of low CHO availability [2,3,4,5]. However, studies have also shown that exercise performance, especially at high intensity, is impaired following LCHF diets [6,7]. It is found that LCHF diets can lead to a lower ability to oxidise exogenous CHO during exercise [8], in which a suppression of pyruvate dehydrogenase may play a role [9]. These effects, together with low muscle glycogen stores, result in reduced training intensity when following a LCHF diet, and can attenuate training-induced improvements in exercise performance [10,11]. 

It has also been suggested that training with low CHO availability may lead to an increased exercise-induced stress response, reflected by higher cortisol levels, and may lead to an attenuated immune response [12]. This might argue against this use of LCHF diets in sports practice, in particular in view of an often already increased risk for upper respiratory tract infections (URTIs) in athletes [13]. The incidence of URTI in athletes is, amongst others, related to low levels of salivary Immunoglobulin A (s-IgA) [13]. This s-IgA is an antibody isotype that is produced locally by B lymphocytes present in mucosal tissues and appears in mucosal secretions such as saliva, thereby protecting against bacteria and viruses entering the body [13]. A shortage of CHO as energy substrate might stimulate cortisol release, inhibiting B-cell immunoglobulin production, resulting in lower s-IgA levels [14]. To our knowledge, only 2 studies investigated the effect of low CHO availability on s-IgA levels [15,16]. 

When it comes to the time-course of effects of CHO restriction on performance and immune status, several knowledge gaps still exist. This prompted us to carry out the current study in which the short-term stress response following switching to a LCHF diet was compared with the longer-term adaptative response. Therefore, in the present study, we investigated the effect on the exercise-induced cortisol, s-IgA and metabolic responses of acute (2 days) and prolonged (2 weeks) adherence to a LCHF diet, compared to a high CHO (HC) diet. Cortisol, s-IgA levels, upper respiratory tract symptoms (URTS), respiratory exchange ratio (RER), circulating metabolites, work output and perceived exertion during exercise were measured in this randomized cross-over dietary intervention study. We hypothesized that a LCHF diet would result in increased exercise-induced cortisol responses, reduced s-IgA levels, and a reduced work output, whether or not in combination with increased perceived exertion.

## 2. Materials and Methods 

### 2.1. Participants

A total of fourteen recreational male athletes participated in this study. They were recruited by contacting local cycling and triathlon clubs and via social media. All trained regularly, at least 4 h per week. Additional inclusion criteria were a BMI between 18.5 and 25 kg/m^2^ and age between 18 and 45 years. Exclusion criteria were: presence of food allergies, chronic illnesses, use of asthma-, anti-inflammatory- and/or immunosuppressive medication. All participants needed to have a hemoglobin concentration >8.5 mmol/L, and they had not donated blood during six weeks prior to the study. 

Study enrolment took place between October 2018 and January 2019. The study was conducted at the Human Nutrition Research Unit, Wageningen University & Research. It was approved by the Medical Ethical Committee of Wageningen University (NL6540408118, ClinicalTrials.gov ID: NCT04019730) and conducted in accordance with the Declaration of Helsinki. All participants gave written informed consent prior to participation.

### 2.2. Study Design

**Figure 1:**
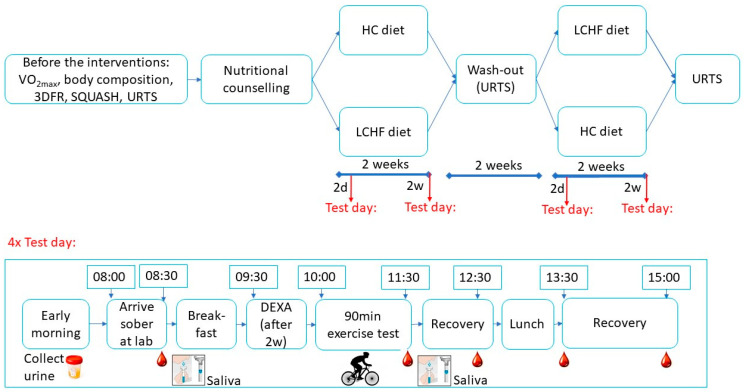
Study design. Schematic study design, 3DRF: 3-day food record; SQUASH: Short Questionnaire to Assess Health enhancing physical activity; URTS: Upper respiratory tract symptoms questionnaire; 2d: after 2 days on the diet; 2w: after 2 weeks on the diet; DEXA: dual energy x-ray absorptiometry.

In a randomized, cross-over design, participants completed two 2-week dietary interventions (Figure 1). General participants characteristics were determined before the start of the first dietary intervention. These included an assessment of maximal aerobic capacity (VO_2max_ test), body composition measurements and questionnaires. Before the intervention, dietary intake was assessed to gain insight in the participants current habitual eating habits and to make an estimation of energy needs. Dietary guidelines were individually explained to participants before the start of each dietary intervention period. Both dietary interventions were followed for 2 weeks. Each dietary intervention period included two exercise test days: one after 2 days on the diet and a second test day after 2 weeks on the diet. The first test day was used to investigate the acute response, and the second test day for the chronic response. Research showed that 5 days adherence to a LCHF diet already resulted in increased fat oxidation [17], therefore we chose to measure after 2 days for a ‘stress response’ from switching to a LCHF diet. A wash-out period, consisting of their habitual diet, of at least two weeks was applied between both diets. Two weeks seemed long enough, as changing to a LCHF diet already leads to adaptations within 5 days [17], and changing back to a HC diet, leads to ‘baseline levels’ after again 5 to 6 days [18]. An upper respiratory tract symptoms (URTS) questionnaire was filled out before the intervention and 2 weeks after each diet ended (Figure 1).

### 2.3. Maximal Aerobic Capacity and Body Composition

A maximal exercise test on a bicycle ergometer (Lode Excalibur, Groningen, The Netherlands) was performed to establish maximal aerobic capacity (VO_2max_). After an initial workload of 100 Watt for 5 min, workload was subsequently increased by either 25 W/min or 40 W/2 min until the participant could not maintain the required pedaling frequency of at least 60 rpm. Participants were allowed to eat and drink before the test; nothing specific was prescribed. Oxygen consumption was measured with indirect calorimetry (Oxycon Carefusion, Hoechberg, Germany), and VO2 max was recorded [19]. Heart rate was monitored by using a heart rate monitor (Polar T31-coded, Oulu, Finland) and connected exercise tracker (Polar FT1). In addition, body length (Seca 213 portable stadiometer, Hamburg, Germany) and weight (Seca 761 scale) were measured. Thereafter, DEXA measurements were carried out using a Lunar Prodigy Advanced DEXA scanner (GE Health Care, Madison, WI, USA) [20]. A quality assurance test was performed to ensure system suitability and precision of the scanner. Whole body scans were performed according to the manufacturer’s protocol and identical scan protocols were used for all subjects.

### 2.4. Dietary Intervention and Physical Activity

Food diaries were obtained before the intervention using a 3-day food record (3DFR) (2 week days and 1 weekend day, randomly assigned). These were analysed for the total energy intake and macronutrient distribution using Compl-eat software ^TM^ (Department of Human Nutrition and Health, Wageningen University, www.compl-eat.nl) [21]. Personalized diet plans were designed based on the participants estimated total energy needs. In total, 6 energy groups were considered: from 10 to 15 MJ with increments of 1 MJ. Participants were instructed to strictly follow their personalized diets. The diets were either a low carbohydrate, high fat diet aiming for ketogenesis (<10 En% Carbohydrates and ~75 En% Fats) or a high carbohydrate diet (~50 En% Carbohydrates and ~35 En% Fats). Protein intake was supposed to be equal in both diets with 15 En%.

Habitual physical activity was assessed before the start of the intervention, using a questionnaire for physical activity level (Short Questionnaire to Assess Health enhancing physical activity (SQUASH)) [22]. Participants were advised to keep their physical activity level the same during both diets, although this was not tracked with a wearable.

### 2.5. Nutritional Counselling

Each participant individually received nutritional counselling. A detailed menu for two weeks and some standard products were provided. For the HC diet these were: 30+ cheese (cheese with less fat per 100 gram), sunflower oil, margarine, nuts, muesli bars, fruit juices. For the LCHF diet these comprised: 48+ cheese (cheese with more fat per 100 g), olive oil, margarine, nuts, low-carb bread and beet muffins. The detailed menu consisted of a shopping list, prescribed recipes for breakfast, lunch, dinner and snacks of every day of the week and information about drinks (water, coffee and tea without sugar or milk were allowed) and herbs. Participants received electronically weighing scales (Impuls, Inter-East B.V., Roosendaal, The Netherlands) to precisely measure their dietary intake to ensure that the prescribed menus were followed during the two weeks of intervention. Participants had to weigh all products, except for bread, which was measured in standardized household portion sizes. Deviations from the diet were written down by the participants and leftovers were measured at the end of both intervention periods to assess compliance. Dietary intake was assessed at the end of each diet by calculating the deviations from the diet that were written down by the participants and by subtracting the leftovers from the provided foods which were weighted by distribution and return.

### 2.6. Exercise Test Days

Test days were performed after 2 days and 2 weeks on each of the diets. See Figure 1 for an overview of the test day. Participants arrived after an overnight fast. At home, they already collected their morning urine to assess ketosis (ketostick, strips 50 A2880 B51, Bayer, Leverkusen, Germany). At 08:00 AM an intravenous cannula was inserted in an antecubital vein and a first blood sample was taken at 08:30 AM. Simultaneously, participants donated saliva via unstimulated, passive drool [23]. A standardized breakfast customized to their energy needs and current diet was provided after the first blood drawing (LCHF breakfast: 588 kCal (average) ~74 En% fat, 17 En% protein, 6 En% carb; HC breakfast: 505 kCal (average) ~34 En% fat, 15 En% protein, 48 En% carb). Thereafter, only after 2 weeks on both diets, body composition was assessed using a DEXA scan (GE Health Care, Madison, WI, USA). Scans were performed on the same time of the day during all sessions to minimize measurement errors.

Next, a 90 min bicycle ergometer test (Lode Excalibur, Groningen, The Netherlands) at 60% of the athletes’ individual W_max_ (~70% VO_2max_) was performed from 10:00 am to 11:30 AM. If an athlete failed to maintain the prescribed workload, the workload was decreased to a level at which the athlete could keep on cycling until the end of the test. Adjustments were written down and the power multiplied by time in seconds was used to calculate total workload. Workload during the 90 min exercise tests was assessed as area under the curve in kilo joule (kJ).

Heart rate was measured with a heart rate belt (Polar T31-coded, Oulu, Finland), placed around the chest. Gaseous exchange was measured (Oxycon Carefusion, Hoechberg, Germany) before the start of the exercise test (while sitting still on the bike for 5 min) and at 60 min during the exercise test during a 5 min period, to assess respiratory exchange rate (RER: ratio VCO_2_/VO_2_).

Participants were allowed to drink plain water during the test, but were not allowed to eat. Drinking was not allowed in the last 10 min of the exercise test. Directly after the exercise test a Borg scale was shown to ask for the rate of perceived exertion (RPE) and another blood sample and saliva sample were taken. Thereafter, participants could take a shower and relax. Another blood sample was taken 1 h after the end of the exercise. Subsequently participants received a standardized lunch customized to their energy needs and current diet (LCHF lunch: 1027 kCal (average) ~78 En% fat, 16 En% protein, 4 En% carb; HC lunch: 781 kCal (average) ~31 En% fat, 14 En% protein, 52 En% carb). Two more blood samples were taken at 2 and 3.5 h after exercise, respectively. These time points were chosen because we aimed to analyse the immunological response more in depth at a later stage. This would include, for example, cytokine responses, and based on their different reaction times, we collected samples at these time points (Figure 1).

### 2.7. Blood Sampling and Analysis

Blood samples were collected in lithium-heparin, EDTA and serum tubes. Lithium-heparin tubes (4.5 mL LH PST^TM^ II, Becton-Dickinson, NJ, America) were centrifuged at 1300 CRF for 10 min at room temperature (RT), plasma was frozen at −80 °C until it was analyzed for glucose concentrations. Glucose was measured by means of an end-point technique (Siemens, The Netherlands). EDTA tubes (8 mL, Becton-Dickinson, NJ, America) were centrifuged at 1200 G for 15 min at 4 °C, and plasma was frozen at −80 degrees until it was analysed for free fatty acids concentrations. Free fatty acids were assessed using an enzymatic test kit according to the manufacturer’s protocol (InstruChemie, Delfzijl, The Netherlands). Serum tubes (5 mL, Becton-Dickinson, NJ, USA) were set aside for at least 30 min, where after they were centrifuged at 1300 G for 10 min at RT, serum was frozen at −80 degrees until it was analysed for ketone content and cortisol concentration. Beta-hydroxybutyrate (βHB) was determined via a colorimetric enzymatic assay (Sigma-Aldrich; St. Louis, MO, USA). Analysis was performed according to manufacturer’s protocol. Cortisol was measured with immunometric chemiluminescence (sandwich) assay with Immulite XPi (Siemens, Den Haag, The Netherlands).

### 2.8. Saliva Sampling and Analysis

Saliva was collected at two time points at every test day: one before breakfast and one directly after exercise. In order to collect whole saliva from the mouth, unstimulated, passive drool was performed [23]. Participants were asked to bend their head slightly downwards and first collect some saliva in their mouth before drooling into the saliva collection aid (Salimetrics, LLC, State College, PA, USA). At least 0.5 mL of saliva was collected in 2-mL collection tubes (Wheaton, Millville, NJ, USA) per time point. Samples were temporarily stored on dry ice and transferred to a refrigerator at −80 °C within seven hours until analysis. IgA antibodies in saliva were determined by enzyme-linked immunosorbent assay (ELISA) as described before [24]. The samples for each individual participant were run on the same assay to eliminate inter-assay variance.

### 2.9. URTS Questionnaires

Before the intervention, and two weeks after the final day of each dietary intervention, participants received a questionnaire about symptoms related to upper respiratory tract infections (URTI). This questionnaire was a Dutch translation of the validated WURSS-21 questionnaire [25].

### 2.10. Statistical Analysis

Data was analysed using IBM SPSS version 27 Statistical Package for Social Sciences (IBM SPSS version 27.0, Armonk, New York, NY, USA). Except for URTS, all data were normally distributed. A paired t-test was performed to assess differences between the LCHF and HC diets. A two-way repeated measures ANOVA (two factor, time x diet) was performed to analyse work, RER, HR and Rate of Perceived Exertion, s-IgA and the cortisol and metabolic response to both diets. When an effect of condition or time or interaction was identified, a pairwise multiple comparison with Bonferroni correction was done to identify the differences. URTS data was analysed using a sign test and the correlation between URTS and s-IgA data was performed using a Spearman correlation test, as data were not normally distributed. The level of significance was set at *p* < 0.05. Data are presented as mean ± SD unless indicated otherwise.

## 3. Results

### 3.1. Participant Characteristics

Baseline characteristics of the fourteen participants are depicted in Table 1. They were active in a variety of sports (cyclist (*n* = 5), triathlete (*n* = 1), climber (*n* = 2), strength trainer (*n* = 2), swimmer (*n* = 1), volleyball player (*n* = 1), football player (*n* = 1), runner (*n* = 1)). They were 32.9 ± 8.2 years old and had a VO2max of 57.3 ± 5.8 mL/kg/min. Their habitual diet contained 2961 ± 528 kCal, 36 ± 6 En% fat, 16 ± 3 En% protein, 43 ± 5 En% carbs. Their habitual training consisted of 5.6 ± 1.1 training hours per week.

### 3.2. Dietary Intake and Blood and Urine Ketone Levels

Energy intake between the LCHF (3104 ± 297 kCal) and the HC diet (3075 ± 298 kCal) was not different (*p* = 0.221). As intended, macronutrient intake was significantly different between both diets, with significantly higher fat intake in the LCHF diet compared to the HC diet (73 ± 1 vs. 33 ± 0 En%, for LCHF and HC, respectively; *p* < 0.001) and, in line with the experimental design, a lower carbohydrate intake in the LCHF diet compared to the HC diet (8 ± 0 vs. 49 ± 0 En%, for LCHF and HC, respectively; *p* < 0.001). Protein intake was higher in the LCHF diet compared to the HC diet (16 ± 1 vs. 15 ± 0 En%, for LCHF and HC, respectively; *p* < 0.001), although this was not intended. An overview of the total daily energy intake and macronutrient distribution at baseline and during both dietary intervention periods, can be seen in Table 2.

The LCHF diet was geared to induce nutritional ketosis. Deviations from the prescribed diets were negligible. Urine ketone levels ranged from 0–1.6 g/L (average: 0.16 ± 0.42 g/L) after 2 days on the LCHF diet and ranged from 0–0.8 g/L (0.26 ± 0.25 g/L) after 2 weeks on the LCHF diet. One out of the 14 participant had no detectable ketones in his urine after 2 weeks on the LCHF diet. There were no ketones present in urine samples during the HC diet. Baseline blood ketone (β-hydroxy-butyrate) levels ranged from 0.06–0.68 mmol/L (average: 0.31 ± 0.18 mmol/L) after 2 days and from 0.21–0.97 mmol/L (0.54 ± 0.26 mmol/L) after 2 weeks on the LCHF diet. On the HC diet, baseline ketone levels were significantly lower: after 2 days ranging from 0.06–0.45 mmol/L (0.14 ± 0.10 mmol/L) and after 2 weeks ranging from 0.05–0.32 mmol/L (0.13 ± 0.08 mmol/L) (*p* < 0.001 compared to the LCHF diet for both test days).

### 3.3. Body Composition

Compared to baseline (76.4 ± 5.4 kg), body mass was significantly lower after 2 weeks on the LCHF diet (74.0 ± 4.5 kg, *p* < 0.001) and after 2 weeks on the HC diet (75.1 ± 4.7 kg, *p* = 0.003). Body mass was also significantly lower after 2 weeks on the LCHF diet compared to body mass after 2 weeks on the HC (*p* = 0.005). Body fat percentage was lower after each of the diets (LCHF: 12.9 ± 4.3% and HC: 13.5 ± 4.6%) compared to baseline (14.5 ± 4.6%, both *p* < 0.001). Body fat percentage was not different between diets (*p* = 0.101). Lean body mass percentage was higher after both diets (LCHF: 82.8 ± 4.2 and HC: 82.2 ± 4.5%) compared to baseline (81.3 ± 4.4%, *p* = 0.017 and *p* = 0.011, respectively). Bone mineral content (BMC) was 4.3 ± 0.3% (3.2 ± 0.2 kg) and comparable between diets (*p* = 0.271). The difference in lean mass percentage between diets was also not significant (*p* = 0.110).

### 3.4. Work output, Respiratory Exchange Ratio and Perceived Exertion

Exercise data can be found in Table 3. The total work in kJ that had to be performed during the 90 min exercise was 1120 ± 148 kJ. However, exercise intensity had to be reduced on multiple occasions. The total work output was significantly lower during the LCHF diet compared to the HC diet, both after 2 days as well as after 2 weeks (939 ± 163 vs. 1042 ± 151 kJ after 2 days and 1003 ± 129 kJ vs. 1043 ± 141 kJ after 2 weeks, for LCHF and HC diet, respectively, *p* < 0.02 between diets). Total workload significantly increased on the LCHF diet after 2 weeks compared to 2 days (*p* = 0.03), while no time-effect was seen for the HC diet. Substrate oxidation patterns at rest and during exercise were significantly different between diets. At rest, RER was significantly lower after 2 days and after 2 weeks on the LCHF diet (0.76 ± 0.03 and 0.77 ± 0.06) compared to the HC diet (0.86 ± 0.05 and 0.87 ± 0.05) (both *p* < 0.001). Additionally, during exercise, RER was significantly lower after 2 days and 2 weeks on the LCHF diet (0.82 ± 0.03 and 0.82 ± 0.03) compared to the HC diet (0.90 ± 0.04 and 0.91 ± 0.02) (both *p* < 0.001). Within each diet group, RER at rest and during exercise did not differ between 2 days and 2 weeks (*p* > 0.05). Heart rate during exercise was significantly higher after 2 weeks on the LCHF diet compared to the HC diet (170 ± 11 bpm vs. 165 ± 13 bpm, *p* = 0.001). There was no significant difference in heart rate between the diets after 2 days (165 ± 13 for LCHF vs. 164 ± 18 for HC, *p* = 0.652). Participants rated their perceived exertion higher after 2 days on the LCHF diet compared to 2 days on the HC diet (18.0 ± 1.4 vs. 15.5 ± 2.7, for LCHF vs. HC; *p* = 0.001). This difference in perceived exertion diminished after 2 weeks, but still tended to be higher on the LCHF diet (17.3 ± 1.7 vs. 16.1 ± 2.0, for LCHF and HC; *p* = 0.053).

### 3.5. Blood Metabolites (Free Fatty Acids, Glucose, Cortisol and Ketone Bodies)

**Figure 2:**
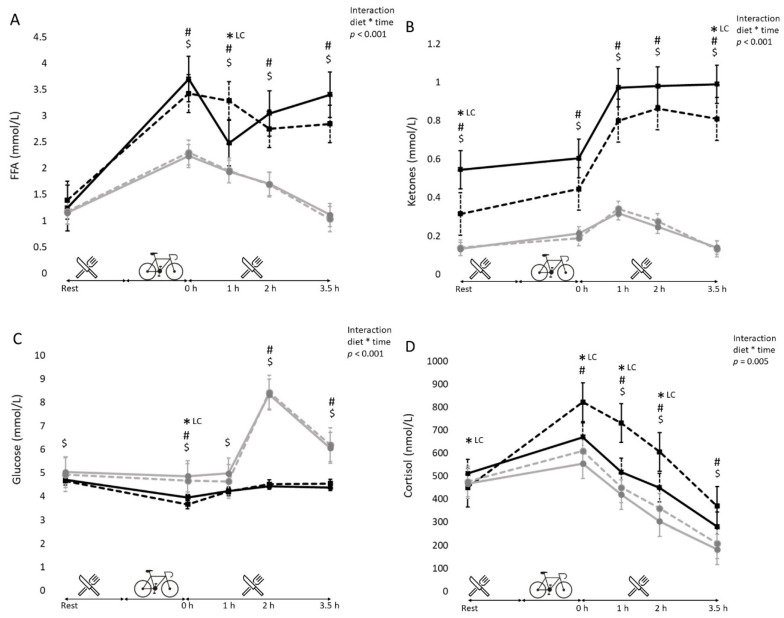
Metabolites. Circulating concentrations of Free fatty acids (**A**), Ketones (**B**), Glucose (**C**) and Cortisol (**D**) measured after 2 days on the LCHF diet (black dotted lines) and HC diet (grey dotted lines) and after 2 weeks on the LCHF diet (black continues line) and HC diet (grey continues line). Means ± SE are shown. All variables showed significant interactions (diet x time) effects. * LC indicates that this difference was between 2 days and 2 weeks on the LCHF diet. Within the HC diet there were no differences between concentrations after 2 days and 2 weeks on that diet. # indicates significant differences between the LCHF and HC diet after 2 days. $ indicates significant differences between the LCHF and HC diet after 2 weeks.

Blood metabolite levels over time are depicted in Figure 2. Circulating markers of lipid metabolism indicated a significantly difference between the HC and LCHF diet. Serum free fatty acids (FFAs) at baseline were comparable between diets and test days (*p* > 0.05). However, peak FFAs levels at the end of the exercise were significantly higher with the LCHF diet (3.4 ± 0.9 and 3.7 ± 0.8 mmol/L after 2 days and 2 weeks, respectively) compared to the HC diet group (2.3 ± 0.6 and 2.2 ± 0.5 mmol/L after 2 days and 2 weeks, respectively, *p* < 0.001 vs. LCHF). Serum beta-Hydroxy-Butyrate (β-HB) levels were significantly higher with the LCHF diet compared to those with the HC diet at all time points, and at both test days (*p* < 0.001).

Glucose levels were in general lower on the LCHF diet compared to those on the HC diet. Baseline glucose levels were not different between diets after 2 days on each of the diets (4.7 ± 0.6 vs. 4.9 ± 0.4 mmol/L for LCHF vs. HC, *p* = 0.153), but were significantly lower after 2 weeks on the LCHF diet (4.7 ± 0.4 vs. 5.0 ± 0.4 mmol/L, for LCHF vs. HC; *p* = 0.035). The exercise-induced decrease in glucose was large on the LCHF diet (−1.00 ± 0.76 mmol after 2 days and −0.76 ± 0.27 mmol/L after 2 weeks, both *p* < 0.001 compared to baseline glucose levels), and much smaller after 2 days on the HC diet (−0.26 ± 0.37 mmol/L, *p* = 0.018 compared to baseline glucose levels) or even absent after 2 weeks on the HC diet (−0.018 ± 0.49 mmol/L, *p* = 0.192). After lunch (2 h after exercise), glucose levels increased with both diets, but to a greater extent on the HC diet (Figure 2C).

The exercise induced cortisol response was highest after 2 days on the LCHF diet compared to 2 weeks on the LCHF diet (822 ± 215 nmol/L vs. 669 ± 243 nmol/L, for 2 days vs. 2 weeks; *p* = 0.004) and compared to the HC diet (609 ± 208 and 555 ± 173 nmol/L, for 2 days and 2 weeks on the HC diet, both *p* < 0.001 vs. LCHF diet). After 2 days on the LCHF diet, cortisol levels increased by 83% post-exercise, compared to only a 31% increase after 2 weeks. On the HC diet, this increase was 28 and 19% after 2 days and 2 weeks of diet intervention, respectively. Resting plasma cortisol concentration was not affected by diet, as there were no differences between baseline cortisol levels between the diets after 2 days and between the diets after 2 weeks. See Figure 2D.

### 3.6. Salivary IgA

**Figure 3:**
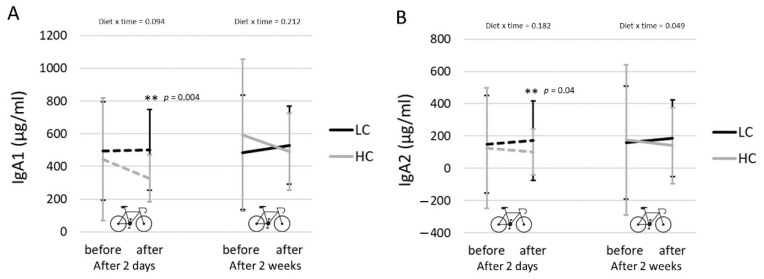
Salivary IgA levels. Salivary IgA1 (**A**) and IgA2 (**B**) levels (mean ± SD) before and after exercise, measured after 2 days on the LCHF diet (black dotted lines) and HC diet (grey dotted lines) and after 2 weeks on the LCHF diet (black continues line) and HC diet (grey continues line). Significant *p* values represent a paired samples t-test for the HC vs. LCHF diet.

No clear exercise effect was seen for salivary IgA1 and IgA2, neither during the HC diet, nor the LCHF diet. See Figure 3. There was a significant interaction between diet and time point (before vs. after exercise) for s-IgA2 after 2 weeks on both diets (*p* = 0.049). Post-exercise s-IgA1 and s-IgA2 levels were lower on the HC diet compared to the LCHF diet after two days adaptation (s-IgA1: 326 ± 143 vs. 502 ± 247 µg/mL for HC and LCHF; *p* = 0.004; s-IgA2: 102 ± 96 vs. 149 ± 162 µg/mL for HC and LCHF; *p* = 0.04).

### 3.7. URTS

The URTS scores for the LCHF and HC diet were 1.8 ± 2.3 and 3.6 ± 5.5, respectively. A sign test did not show any statistically significant difference between the two median URTS scores (*p* = 0.187). After both diets, all participants rated the question “how ill do you feel today?” with a 0 “not ill” or a 1 “very mildly” on a scale of 0 to 7 “very ill”. For the LCHF diet, only one participant rated this question with a 1, all other participants rated this question with a 0. For the HC diet, 3 participants rated this question with a 1, all others with a 0. In addition, there were no significant correlations between URTS and s-IgA levels, *p* > 0.05.

## 4. Discussion

We aimed to investigate short-term (2 days) and prolonged (2 weeks) effects of adherence to a LCHF diet with regard to its effects on exercise-induced cortisol, s-IgA and metabolic responses, and compared this to a HC diet. This is because, to our knowledge, studies of the time course of effects after a low carbohydrate diet are relatively scarce, but relevant. We showed that the LCHF diet resulted in a reduced work output and a higher perceived exertion both after 2 days and 2 weeks, in addition to marked metabolic differences and a pronounced exercise-induced cortisol response after 2 days.

Metabolic effects, work output and perceived exertion: Two days on the LCHF or HC diet resulted in different metabolic effects during the exercise trial. After cycling, participants following the LCHF diet showed higher circulating FFA and ketone levels, whereas their plasma glucose levels and RERs were lower at that time point, indicating more reliance on fat oxidation in comparison to the HC diet. Except for a further increase in ketone levels with the LCHF diet, these differences were similar after 2 weeks on the different diets. Plasma free fatty acids at baseline were not different between diets and between days, which can be explained by a lower release of FFA from the liver and adipose tissue during the LCHF diet. Free fatty acids peaked after exercise in the LCHF diet. Plasma FFAs also increased after exercise in the HC diet, which is in agreement with other studies [6,26]. During exercise, the rate of lipolysis increases and, as a result, the concentration of free fatty acids in plasma increases [27]. The higher plasma ketone levels at baseline in the LCHF diet confirm limited CHO availability after 2 days. Apparently, this was not visible yet from blood glucose levels, which were only lower after 2 weeks on the LCHF diet. A decrease of blood glucose levels after exercise following a LCHF diet has also been observed in other studies [6]. The marked glucose peak observed in the HC diet group after consuming the standardized meal was expected as this meal contained ~200 g of carbohydrates.

The reduced work output after following the LCHF diet for 2 days is in line with previous studies and can be explained by decreased CHO oxidation, even though fat oxidation rates may already be increased after short term adaptation [18]. This was emphasized by the higher rates of perceived exertion. Although after 2 weeks on the LCHF diet the work output was higher than after 2 days, suggesting some adaptation towards improved fat oxidation, it was still lower compared to that following the HC diet. At the same time, heart rate was higher and perceived exertion equal between both test days on the LCHF diet. The lower RER after 2 days on the LCHF diet observed in our study is in agreement with other findings suggesting that increased fat oxidation can occur within days during low carbohydrate availability [18,28]. Studies suggest that prolonged adherence to a LCHF diet enhances the breakdown, transport, and oxidation of fat in skeletal muscle [29]. However, this was not reflected by a further decrease of the RER in our study. It should be noted that the interpretation of RER, VO2 and VCO2 values for fat and glucose oxidation requires some caution, as the oxidation of ketone bodies confounds the results [30]. Given the higher ketone levels after 2 weeks on the LCHF diet and the slightly improved work output and lower RPE this might play a role in our study as well. It remains speculative whether longer adherence to the LCHF diet would have resulted in smaller differences in work output with those after the HC diet. It has been reported that adaptation to a non-ketogenic low carbohydrate diet would be around 5 days, without further enhancement thereafter [17]. Others have suggested that consumption of a LCHF diet results in adaptations of the homeostatic regulation of muscle glycogen and even further improved fat oxidation during exercise on a longer term [26].

Cortisol levels: Baseline cortisol levels were comparable between both diets, which is in agreement with a study showing no association between resting cortisol levels and any dietary parameter [31]. The marked effect on the exercise-induced cortisol response after 2 days of following a LCHF diet is likely related to the low CHO availability, which is only partly compensated by increased use of fat as a substrate. In that situation, exercise will rapidly lead to glycogen depletion [32] resulting among others in increased cortisol release [33,34]. The exercise intensity in our study was fairly high, reflected by heart rates above 86% HRmax. Several studies have shown that when individuals perform exercise after several days on very low carbohydrate diets, cortisol levels are markedly higher than with a normal or high carbohydrate diet [35,36]. Interestingly, we found that this difference in cortisol response, compared to after a HC diet, was diminished after two weeks. This suggests further adaptation to the LCHF diet.

s-IgA levels: Salivary IgA1 and IgA2 were both lower post-exercise after 2 days on the HC diet compared to levels after 2 days on the LCHF diet, which might suggest that a short-term LCHF diet attenuates exercise-induced decreases in s-IgA. The reduced s-IgA (both in IgA1 and IgA2) is often linked to an increased risk for URTI [37], despite being also debated [38]. There were no differences in post-exercise s-IgA levels after two weeks with both diets, which is in agreement with a 3-week trial, showing that post-exercise changes in s-IgA were comparable between a HC and ketogenic diet [15]. On the other hand, 70% higher s-IgA secretion rates were reported after a 31-day ketogenic diet compared to secretion rates before this ketogenic diet [16]. On beforehand, we expected lower s-IgA levels with the LCHF diet, because higher cortisol levels can result in lower immunoglobulin production by B-cells, thereby attenuating the s-IgA levels [14]. Some researchers suggest that other factors besides cortisol affect s-IgA levels after exercise, for example increased sympathetic nervous system innervation of the salivary glands or total energy availability [16]. In addition, variation in s-IgA levels was very large between our participants, which can be explained by variation in the health status of the oral cavity [38], as well as by variation in sleep practices, psychological stress and flow rate. Unfortunately, we did not assess salivary flow rate, but only s-IgA concentrations, so direct comparisons with these findings are not possible.

In the majority of studies, no separate detection of s-IgA1 and s-IgA2 levels was performed and given the different susceptibility of these isotypes for proteolysis, this might affect the association with exercise-induced changes in mucosal immunity. Both our findings on s-IgA1 and s-IgA2 connected to this apparent discrepancy with cortisol levels merit further investigation, preferably in a long-term study.

Our comparable s-IgA levels after 2 weeks of LCHF and HC diet were also reflected by finding no differences in URTS in our athletes 2 weeks after the end of each of the diets whereby none of the participants indicated to feel ill. Although it should be noted that these URTS questionnaires are filled in by the participants and not established by an additional throat swab. Unfortunately, the data in this study does not suggest that one of the diets could protect against URTS, although the inhibition of the s-IgA decrease after exercise on the LCHF diet after 2 days seems promising. Several articles already stated that there was no evidence of a beneficial effect of carbohydrates on URTS [39,40]. However, whether a LCHF ketogenic diet would have beneficial effects should be studied in future.

Body mass: Mean body mass was lower after following both diets compared to before the intervention. However, these decreases in body mass were expected after the LC diet [41,42], as a LC diet decreases glycogen concentrations, which is associated with a loss of body water and thus body weight [43]. Decreases on the HC diet were not directly foreseen, but may have been caused by underreported energy intakes in the 3-day food records at intake, leading to a dietary advice with a lower energy intake then needed for the participant. This would account for both the HC diet and the LC diet, as athletes were subscribed the same energy group with both diets. This underreporting is common in the athletic population [44]. We don’t think that these changes have affected our results significantly.

Limitations and strengths: To our knowledge, this is the first study applying a cross-over design in which effects of implementing a LCHF diet were measured after 2 days and 2 weeks adherence to the diet, which not only enabled us to compare effects of a LCHF diet with those of a HC diet, but also gain insight in the time-course of these effects.

A limitation is that we did not take salivary flow rate and salivary volume into account, but only salivary IgA concentrations. Although salivary IgA concentrations alone also provide relevant insight, this information would have added to the study since when flow rate and/or volume is low, this might impair mucosal immunity as well.

For some participants, we had to reduce the workload during the exercise sessions, especially during the LCHF test days. This led to a lower work output when participants were on the LCHF diet, which in turn makes it harder to directly compare exercise-induced cortisol responses, metabolites and s-IgA between the diets. On the other hand, perceived exertion and heart rate were higher during the exercise tests during LCHF intervention, which made us conclude that the effort was higher when on the LCHF diet.

In addition, it would have been useful to have additional baseline data for body composition before the start of the second dietary intervention. However, in order to limit the burden of the participants, we had to make decisions on which measurements to include and exclude.

The wash-out period of 2 weeks between the interventions was based on previous findings regarding adaptation time to a LCHF diet [17] and turning back to baseline after CHO loading [18]. Further studies are warranted to explore the time-course of this reversal of effects. In addition, diets were not controlled during the wash-out period, which could have affected our results on the first test day in the second diet. The same holds true for the dietary habits of the participants prior to our study. It has been found that regular consumption of specific food products is associated with differences in exercise-induced muscle damage and cardiac stress [45], which might have influenced cortisol response and perceived exhaustion. Next to this, it is recommendable to repeat the study in female athletes, as they are underreported in research.

In conclusion, the results of the present study showed that 2 days adherence to a LCHF diet already leads to metabolic changes, as reflected by lower RER, lower glucose, higher FFA and higher ketone levels. These metabolic changes were comparable between 2 days and 2 weeks adherence to the LCHF diet, except for ketone levels which were further increased after 2 weeks. On the other hand, the exercise-induced cortisol response was higher after 2 days and attenuated after 2 weeks. The exercise capacity after adherence to the LCHF diet was low, with lower workload, higher or comparable HR and higher RPE compared to the HC diet. A drop in s-IgA following exercise was not seen after 2 days on the LCHF diet, in contrast to the HC diet, which might suggest some form of protective effect, although we could not relate this to URTS. Our results underline that adaptation to a LCHF diet in terms of the metabolic and exercise-cortisol response have different time spans.

## Figures and Tables

**Table 1:** Participant characteristics.

	Participants (*n* = 14)
Age (years)	32.9 ± 8.2
Body composition	
Height (cm)	181.7 ± 4.7
Weight (kg)	76.4 ± 5.4
BMI (kg/m^2^)	23.1 ± 1.4
Lean mass (kg)	61.9 ± 3.4
Lean mass (%)	81.3 ± 4.4
BMC (kg)	3.2 ± 0.25
BMC (%)	4.2 ± 0.32
Body fat (kg)	11.2 ± 4.0
Body fat (%)	14.5 ± 4.6
Total training (hours/week)	5.6 ± 1.1
Maximal exercise performance	
VO2max (ml/kg/min)	57.3 ± 5.8
Max heart rate (bpm)	187 ± 9
Max Power (Watt)	346 ± 46
Max Power/kg body weight	4.5 ± 0.5

Means ± SD are shown. BMI: Body mass index; BMC: Bone mineral content. Physical characteristics are determined during a VO2max test.

**Table 2:** Dietary intake and fasting serum and urine ketone levels.

	Habitual	LCHF Diet	HC Diet	*p* Value
Energy (kCal)	2961 ± 528	3104 ± 297	3075 ± 298	0.221
Protein (g/day)	116 ± 22	124 ± 12	112 ± 11	<0.001
Protein (En%)	16 ± 3	16 ± 1	15 ± 0	<0.001
Carbohydrate (g/day)	318 ± 72	64 ± 6	373 ± 38	<0.001
Carbohydrate (En%)	43.4 ± 5.3	8 ± 0	49 ± 0	<0.001
Total Fat (g/day)	122 ± 29	254 ± 25	116 ± 11	<0.001
Total Fat (En%)	36 ± 6	73 ± 1	33 ± 0	<0.001
Saturated Fat (g/day)	43 ± 13	68 ± 6	32 ± 3	<0.001
Saturated Fat (En%)	13.1 ± 3.2	19.7 ± 0.3	9.3 ± 0.3	<0.001
Monounsaturated Fat (g/day)	46 ± 13	127 ± 13	35 ± 3	<0.001
Monounsaturated Fat (En%)	13.9 ± 3.3	36.8 ± 1.1	10.3 ± 0.2	<0.001
Polyunsaturated Fat (g/day)	22 ± 7	39 ± 5	41 ± 5	0.002
Polyunsaturated Fat (En%)	6.6 ± 1.8	11.4 ± 0.4	12.1 ± 0.2	<0.001
Cholesterol (mg/day)	354 ± 242	699 ± 57	165 ± 18	<0.001
Dietary Fiber (g/day)	31 ± 6	28 ± 3	41 ± 4	<0.001
Dietary Fiber (En%)	2 ± 0	5 ± 1	9 ± 2	<0.001
Fasting serum βHB (mmol/L)		0.27 ± 0.13	0.07 ± 0.04	<0.001
Urine ketone levels (g/L)		0.26 ± 0.25	0.00 ± 0.00	<0.001

Means ± SD are shown. *p*-values represent a dependent t-test between both intervention diets (LCHF vs. HC); serum βHB and urine ketone bodies represent data after following the diets for 2 weeks.

**Table 3:** Work, RER, HR and RPE.

	LCHF			HC			Intervention	
			Time Effect			Time Effect	after 2d	after 2w
	after 2 Days	after 2 Weeks	*p*-Value	after 2 Days	after 2 Weeks	*p*-Value	*p*-Value	*p*-Value
Work (AUC in kJ)	939 ± 163	1003 ± 129	0.030	1042 ± 151	1043 ± 141	0.974	0.004	0.016
RER (rest)	0.76 ± 0.03	0.77 ± 0.06	0.282	0.86 ± 0.05	0.87 ± 0.05	0.564	<0.001	<0.001
RER (at t60)	0.82 ± 0.03	0.82 ± 0.03	0.681	0.90 ± 0.04	0.91 ± 0.04	0.612	<0.001	<0.001
HR (in bpm; at t60)	165 ± 13	170 ± 11	0.014	164 ± 18	165 ± 13	0.633	0.652	0.001
RPE score	18.0 ± 1.4	17.3 ± 1.7	0.151	15.5 ± 2.7	16.1 ± 2.0	0.300	0.001	0.053

Values are mean ± SD, calculated after 2 days and 2 weeks on both diets. LCHF: low carbohydrate high fat diet; HC: high carbohydrate diet; AUC: area under the curve; kJ: kilo Joule; RER: respiratory exchange ratio; t60: after 60 min exercise; HR: heart rate; RPE: rate of perceived exertion. *p*-values represent repeated measures ANOVA.

## Data Availability

The data presented in this study are available on request from the corresponding author. The data are not publicly available due to ethical reasons

## Declaration

The results of the present study do not constitute endorsement by ACSM. We declare that the results of this study are presented clearly, honestly, and without fabrication, falsification, or inappropriate data manipulation.
